# MIDDAS-M: Motif-Independent *De Novo* Detection of Secondary Metabolite Gene Clusters through the Integration of Genome Sequencing and Transcriptome Data

**DOI:** 10.1371/journal.pone.0084028

**Published:** 2013-12-31

**Authors:** Myco Umemura, Hideaki Koike, Nozomi Nagano, Tomoko Ishii, Jin Kawano, Noriko Yamane, Ikuko Kozone, Katsuhisa Horimoto, Kazuo Shin-ya, Kiyoshi Asai, Jiujiang Yu, Joan W. Bennett, Masayuki Machida

**Affiliations:** 1 Bioproduction Research Institute, National Institute of Advanced Industrial Science and Technology (AIST), Sapporo, Hokkaido, Japan; 2 Bioproduction Research Institute, National Institute of Advanced Industrial Science and Technology (AIST), Tsukuba, Ibaraki, Japan; 3 Computational Biology Research Center, National Institute of Advanced Industrial Science and Technology (AIST), Koto-ku, Tokyo, Japan; 4 Japan Biological Informatics Consortium, Koto-ku, Tokyo, Japan; 5 Molecular Profiling Research Center for Drug Discovery, National Institute of Advanced Industrial Science and Technology (AIST), Koto-ku, Tokyo, Japan; 6 Biomedical Research Institute, National Institute of Advanced Industrial Science and Technology (AIST), Koto-ku, Tokyo, Japan; 7 Beltsville Agricultural Regional Research Center, Agricultural Research Service, United States Department of Agriculture, Beltsville, Maryland, United States of America; 8 Department of Plant Biology and Pathology, Rutgers University, New Brunswick, New Jersey, United States of America; Woosuk University, Republic of Korea

## Abstract

Many bioactive natural products are produced as “secondary metabolites” by plants, bacteria, and fungi. During the middle of the 20th century, several secondary metabolites from fungi revolutionized the pharmaceutical industry, for example, penicillin, lovastatin, and cyclosporine. They are generally biosynthesized by enzymes encoded by clusters of coordinately regulated genes, and several motif-based methods have been developed to detect secondary metabolite biosynthetic (SMB) gene clusters using the sequence information of typical SMB core genes such as polyketide synthases (PKS) and non-ribosomal peptide synthetases (NRPS). However, no detection method exists for SMB gene clusters that are functional and do not include core SMB genes at present. To advance the exploration of SMB gene clusters, especially those without known core genes, we developed MIDDAS-M, a motif-independent *de novo* detection algorithm for SMB gene clusters. We integrated virtual gene cluster generation in an annotated genome sequence with highly sensitive scoring of the cooperative transcriptional regulation of cluster member genes. MIDDAS-M accurately predicted 38 SMB gene clusters that have been experimentally confirmed and/or predicted by other motif-based methods in 3 fungal strains. MIDDAS-M further identified a new SMB gene cluster for ustiloxin B, which was experimentally validated. Sequence analysis of the cluster genes indicated a novel mechanism for peptide biosynthesis independent of NRPS. Because it is fully computational and independent of empirical knowledge about SMB core genes, MIDDAS-M allows a large-scale, comprehensive analysis of SMB gene clusters, including those with novel biosynthetic mechanisms that do not contain any functionally characterized genes.

## Introduction

Chemists have been deciphering the chemical structures of natural products for a century and a half. Many of these natural products are produced as “secondary metabolites” by plants, bacteria, and fungi. During the middle of the 20^th^ century, several secondary metabolites from fungi revolutionized the pharmaceutical industry. These include the antibiotic, penicillin; the cholesterol-level lowering compound, lovastatin; and the immune suppressor, cyclosporin. Other fungal secondary metabolites have achieved notoriety, such as aflatoxin [1]. In the late 20^th^ century, with the advent of gene cloning, it became apparent that fungal secondary metabolites are biosynthesized by clusters of coordinately regulated genes. Such gene clustering is rare in eukaryotes.

In spite of limited number of secondary metabolites identified from a single species, sequencing the genomes of filamentous fungi has revealed far more than the expected numbers of secondary metabolite biosynthetic (SMB) genes. The numbers of SMB genes encoding polyketide synthases (PKSs) and non-ribosomal peptide synthetases (NRPSs) range from 17–35 and 14–24, respectively, in the individual genomes of eight *Aspergillus* species [2]. To identify potential secondary metabolites (SMs) in filamentous fungi, various bioinformatics tools, including SMURF [3], antiSMASH [4], [5], CLUSEAN [6], and the method described by Andersen et al. [7], have been developed and successfully applied. The basic concept underlying these tools is the existence of SMB gene clusters, which typically contain approximately 20 genes, including the so-called core genes of PKS, NRPS, or dimethylallyl tryptophan synthases (DMATs). These methods are completely dependent on the known sequence motifs of the core genes; therefore, they can only be used to detect SMB gene clusters that include these core genes. In addition, they cannot distinguish functional clusters from silent or cryptic clusters in fungi [8] because they do not incorporate transcriptomics data.

Many secondary metabolites with important medicinal activities have scaffold structures that are mostly synthesized by the core genes of PKS or NRPS, but there are also others independent of those core genes such as oxylipins, a derivative of fatty acids [9]. We recently discovered the SMB gene cluster for kojic acid (KA), which is the representative secondary metabolite of *Aspergillus oryzae* [10], [11]. The KA cluster could not be detected by conventional methods due to the lack of the core genes. KA was discovered in 1907 and has been used industrially [12], but its biosynthetic gene cluster was found only recently. This fact indicates the extreme difficulty in identifying SMB gene clusters without any core genes.

Comparative genomics has shed light on the characteristics of SMB genes that localize to so-called non-syntenic blocks (NSBs) [13]–[15]. NSBs harbor genes that have roles in the transport and metabolism of various compounds [13] and are highly divergent between species [16]–[18]. Two-thirds of the genes in NSBs are not homologous with any genes with known functions [13]. Considering our limited knowledge regarding SMB genes and their high level of diversity, it can be speculated that the significant accumulation of unknown genes on NSBs is due to the presence of a large number of SMB genes on NSBs. In support of this hypothesis, the KA gene cluster is located in an NSB [11].

To enhance the exploration of SMB gene clusters in fungal genomes, especially those without core genes, we have developed MIDDAS-M, a motif-independent *de novo* detection algorithm for secondary metabolite gene clusters. We used virtual gene cluster generation on an annotated genome sequence integrated with highly sensitive and accurate scoring for the cooperative transcriptional regulation of cluster member genes. MIDDAS-M accurately predicted 38 SMB gene clusters in 3 fungal strains that have been experimentally confirmed and/or predicted by other motif-dependent methods. In addition, we discovered a novel SMB cluster with a potentially new mechanism of cyclic peptide biosynthesis using MIDDAS-M. The cluster was experimentally validated to perform ustiloxin B biosynthesis. Because it is fully computational and independent of empirical knowledge about SMB core genes, MIDDAS-M permits a large-scale, comprehensive analysis of SMB gene clusters, including those with novel biosynthetic mechanisms that do not contain any functionally characterized genes.

## Results

### MIDDAS-M algorithm

**Figure 1:**
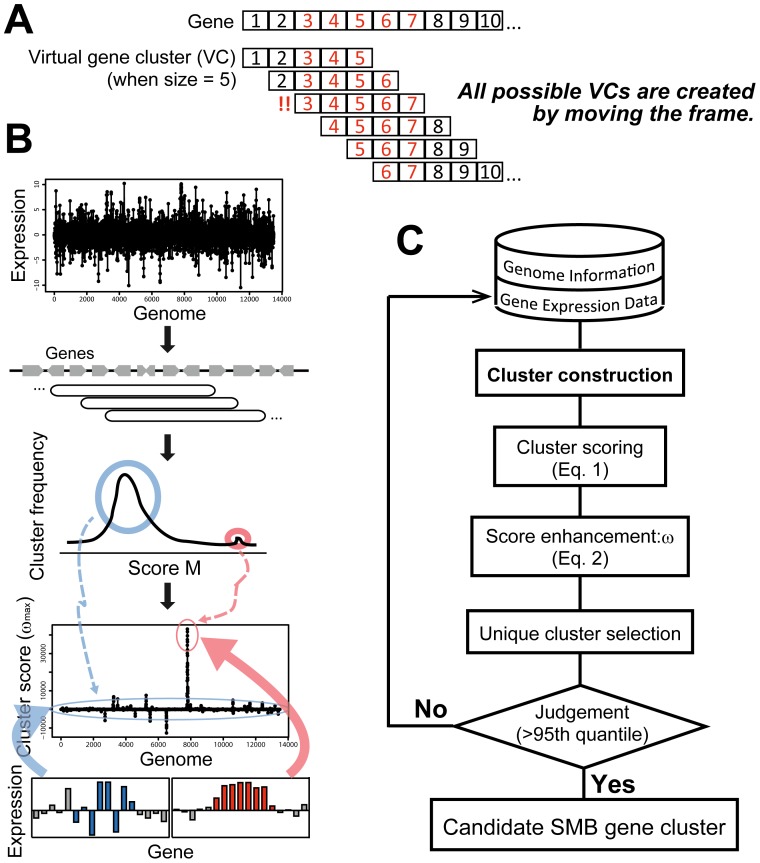
Principle of the MIDDAS-M algorithm. (A) Virtual cluster (VC) generation for SMB gene cluster detection. Gene clusters on a genome are evaluated comprehensively by a moving window with a specific cluster size; the cluster size can be changed from 3 to 30 or another appropriate size. (B) Schematic representation of MIDDAS-M. Candidate SMB gene clusters show large deviations from the standard deviation after summing the induction ratios of member genes and statistical enhancement. (C) Flow chart of the MIDDAS-M algorithm.

The algorithm depends on the concurrent expression of SMB cluster member genes. First, all possible gene clusters (virtual clusters, VCs) are identified in a previously gene-annotated genome by moving a frame with a given cluster size (*ncl*) from 3 to 30 genes (Fig. 1A). The cluster induction ratio (*M* score) for a VC is calculated by summing the induction ratios of all genes in the VC. For a given gene, the induction ratio is determined by dividing the expression level of the gene in an SM-producing condition by the expression level in a non-SM-producing condition. The *M_i,ncl_* score for each VC, which begins at gene *i* with cluster size *ncl*, was determined according to the following equation:
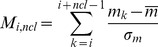
(1)where *m_k_* is the induction ratio of gene *k*, and 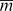 and σ*_m_* are the mean and the standard deviation of all *m* values, respectively. As shown in Equation 1, each *m* value should be normalized by *Z*-score transformation before the summation. *M* scores are evaluated for each *ncl* from 3 to an appropriate upper limit (30 in this study). Using this procedure, the *M* scores of “non-real” clusters in which genes are not co-regulated should have low absolute values because positive values are cancelled out by negative values, and vice versa. In contrast, *M* scores of “real” SMB clusters show significantly high absolute values because the genes in the cluster are regulated concurrently (Fig. 1B).

**Figure 2:**
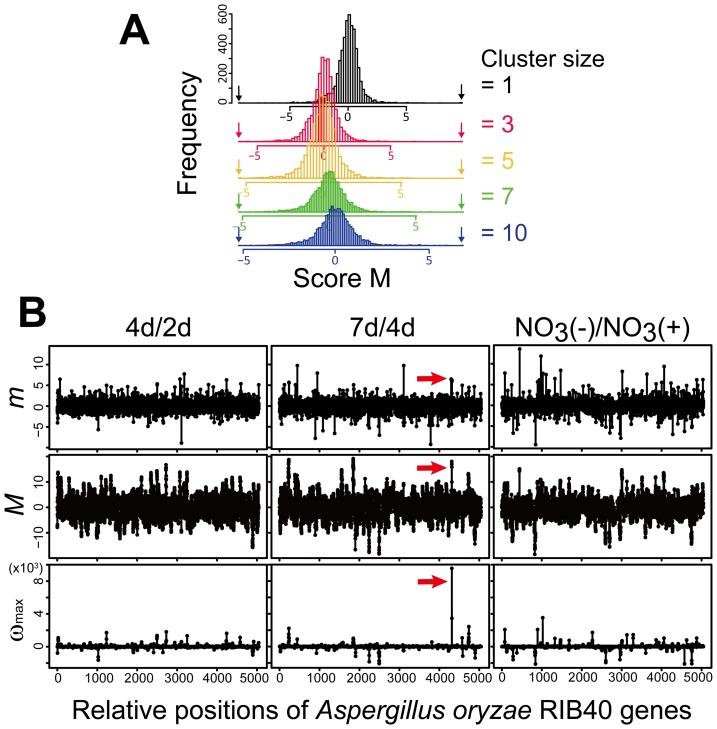
Behavior and performance of MIDDAS-M in *A. oryzae*. (A) Histograms of *M* scores at *ncl* = 1, 3, 5, 7, and 10 in the transcriptomes at 7 vs. 4 days of cultivation in kojic acid (KA)-production medium. The symmetry broke at a cluster size of 3 because of the emergence of large *M* scores due to the induction of the KA cluster genes. Arrows at the termini of the *x*-axis indicate the smallest and the largest values. (B) Emergence of a ω_max_ peak by MIDDAS-M from the raw induction ratio. The *x*-axis designates relative position of the genes on the *A. oryzae* RIB40 genome when eight chromosomes are concatenated into one. The *y*-axis scales are the same for all three datasets in the same raw. The ω_max_ peak indicated by the red arrow corresponds exactly to the three genes responsible for KA production.

SMB cluster candidates exhibit relatively high *M* scores, but the background noise from pseudo-positive VCs remains high (Fig. 2B). To help distinguish between VCs that are SMB clusters and those that are not, *M* scores deviating from the normal distribution are magnified by statistical treatment. The magnified score, *ω_i,ncl_*, was evaluated for each *M_i,ncl_* at each *ncl* using the following equation:
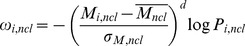
(2)where 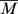 *_ncl_* and *σ_M,ncl_* are the mean and the standard deviation, respectively, of all *M* scores at *ncl*, *d* is a positive odd integer as an order of the moment (set as 3 in this study), and *P_i,ncl_* is the occurrence probability of *M_i,ncl_* in the distribution of all *M* scores at *ncl*. The moment expresses the magnitude of deviation from standard distribution, being emphasized as the order *d* increases. An SMB cluster candidate with *M_i,ncl_* largely deviated from the mean value shows a large absolute value of *ω_i,ncl_*, because of the large *Z*-score (the content in the parenthesis of Equation 2) and the logarithmic *P_i,ncl_* (<<1) converging to minus infinity. The *ω* score shows a positive or negative value when the gene cluster is induced or repressed, respectively.

For each starting gene, the *ncl* showing the largest ω value (ω_max_) is chosen as the cluster size. This step contributes to the high sensitivity of MIDDAS-M by surveying clusters of different sizes. Finally, the clusters showing the largest ω_max_ among overlapping VCs (sub-clusters of a candidate cluster) are defined as the “unique” cluster (detailed explanation with an example is described in the “MIDDAS-M computation” section of the Supplementary Method in Appendix S1). MIDDAS-M also automatically generates the candidate clusters from all possible pairwise comparisons of transcriptomes from several or more culture conditions. This allows comprehensive *de novo* predictions using large-scale transcriptome datasets based on a variety of culture conditions. See Supplementary Method, the “MIDDAS-M computation” section in Appendix S1 for further details. MIDDAS-M is available for use at the following server (http://133.242.13.217/MIDDAS-M).

### Accurate detection of experimentally validated SMB gene clusters

MIDDAS-M was applied to the filamentous fungus *A. oryzae* for the detection of the KA gene cluster. This metabolite is an inhibitor of pigment formation in animal tissues and is therefore used as a skin-whitening compound in cosmetics [19], [20]. The KA cluster was recently found to be composed of only three genes, none of which encodes a PKS, NRPS, or other core SMB enzyme. Instead, the three genes encode an oxidoreductase, a Zn(II)_2_-Cys_6_ (C6)-type transcription factor, and a major facilitator superfamily transporter [10], [11]. KA production is typically observed after 3 to 4 days of inoculation of *A. oryzae* in liquid growth media, and can be stopped by adding a small amount of sodium nitrate to the medium [21], [22].

Figure 2 shows the results of MIDDAS-M analysis for three *A. oryzae* transcriptomes in the relative transcription observed under KA-inducing vs. KA-non-inducing conditions in two-color DNA microarray experiments; 4 vs. 2 days, 7 vs. 4 days, and without vs. with nitrate. Among the 12,084 genes of *A. oryzae* [13], 5,046 genes with expression in all three datasets were used for the analysis. The *M* scores for the 7/4-day dataset are normally distributed when the cluster size *ncl* = 1, but the symmetry was lost, and the top of the distribution slid to the left, when *ncl* = 3 and 5, accompanied by the emergence of large *M* scores outside of the normal distribution (Fig. 2A). MIDDAS-M emphasizes this deviation of the SMB cluster candidates through Equation 2, enabling their sensitive detection. In the 7/4-day dataset, a distinct single peak emerged in the ω_max_ score from the gene induction ratio (*m* value) as designated by a red arrow in Fig. 2B. The gene cluster corresponding to this peak was composed of three genes, AO090113000136, AO090113000137, and AO090113000138, which were exact matches to the three KA biosynthetic genes [10], [11]. The highly sensitive and specific detection of the KA gene cluster, which has a small cluster size of 3 and does not include any core genes, indicates that MIDDAS-M has strong potential as a motif-independent predictor of SMB gene clusters. In the 4/2-day and without/with nitrate datasets, only small ω_max_ signals were observed, indicating that the increase of KA productivity in the two datasets was not due to the transcriptional induction of the genes responsible for KA biosynthesis.

**Figure 3:**
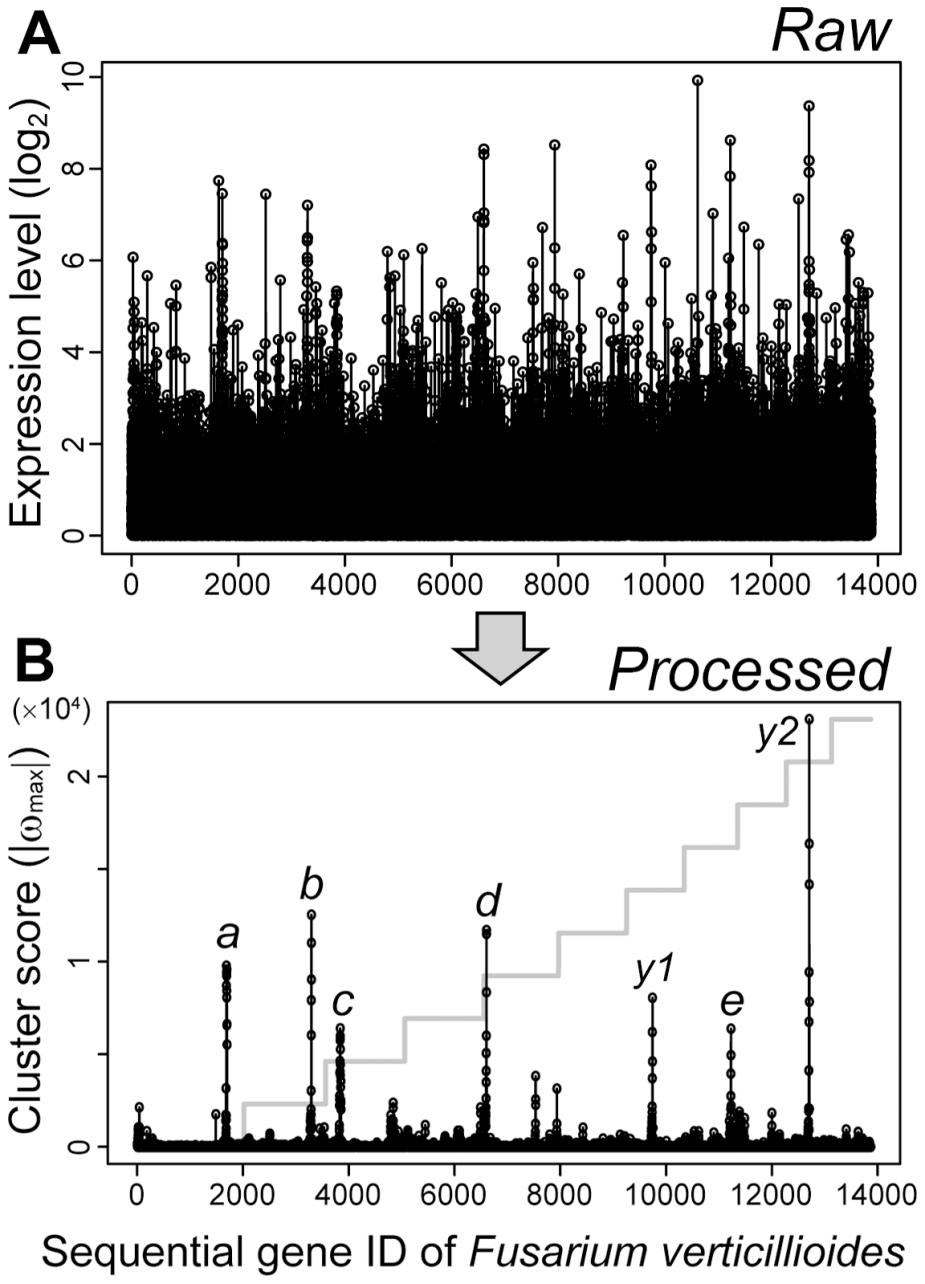
Clear detection of known SMB gene clusters in *F. verticillioides* by MIDDAS-M. (A) Expression levels of each gene on the *F. verticillioides* genome in 4 samples of a transcriptome time series at 24, 48, 72, 96 h in liquid fumonisin-inducing media. The highest value of the 4 expression levels was plotted for each gene. (B) Absolute maximum cluster scores (|ω_max_|) by the comprehensive pair-wise calculation (_4_C_2_) for each gene detected from the same transcriptome data as A. The step line plot in gray denotes the individual chromosomes. The peaks designated by *a* through *e* correspond to the 5 experimentally validated SMB clusters: *a*, fumonisin; *b*, perithecium pigment; *c*, fusaric acid; *d*, bikaverin; *e*, fusarin. Two peaks to which any known gene clusters do not correspond were designated as *y1* and *y2*.

MIDDAS-M was also tested for *Fusarium verticillioides* using a time series of four transcriptomes obtained from mycelia grown in the liquid medium used to induce fumonisin production [23]. This fungus is a plant pathogen that produces mycotoxins and is phylogenetically distantly related to *Aspergillus*. A comprehensive comparison of the 4 transcriptomes followed by the MIDDAS-M prediction yielded several distinct peaks of ω_max_, of which 5 corresponded to the known SMB gene clusters for fumonisin [24], perithecium pigment [23], fusaric acid [23], bikaverin [25], and fusarin [23] (Fig. 3). Although the size of the predicted SMB gene cluster for fusaric acid was three-fold larger than the experimentally validated clusters, the others were almost correct in size (Table 1). This result clearly illustrates the high sensitivity of MIDDAS-M in detecting functional SMB clusters.

**Table 1:** Experimentally-validated or SMURF-annotated SMB gene clusters detected by MIDDAS-M.

Fungus	Compound/SMURF^a^	*ω_max_*	Gene ID^b^	Cluster size		Source
				MIDDAS-M^c^	Other^d^	
*A. oryzae*	Kojic acid	9544	AO090113000136 - AO090113000138	3	3	[10], [11]
*F. verticillioides*	Bikaverin (Cluster 7)	11708	FVEG_03379 – FVEG_03383	4	6	[25], SMURF
	Fumonisin (Cluster 3)	9780	FVEG_00316 – FVEG_00329	14	15	[24], SMURF
	Fusaric acid (Cluster 27)	6398	FVEG_12519 – FVEG_12535	17	5	[23], SMURF
	Fusarin	840	FVEG_11078 – FVEG_11086	9	9	[23], SMURF
	Perithecium pigment (Cluster 9)	12533	FVEG_03696 – FVEG_03699	6	4	[23], SMURF
	Cluster 10	1700	FVEG_05526 – FVEG_05530	5	10	SMURF
	Cluster 24	866	FVEG_11927 – FVEG_11931	5	7	SMURF
*A. flavus*	Aflatoxin (Cluster 54)	99087	AFLA_139150 - AFLA_139320	18+5	29	[37], SMURF
		24302	AFLA_139370 – AFLA_139410			
	Aflatrem	3670	AFLA_096380 - AFLA_096400 (ATM1)	3	3	[44]; Blastn, E0.0
	(Cluster 14)	8984	AFLA_045490 - AFLA_045540 (ATM2)	6	5	
	Cyclopiazonic acid	36281	AFLA_139460 – AFLA_139490	4	3	[45]
	Gliotoxin-like (Cluster 22)	32872	AFLA_064380 – AFLA_064590	22	26	Annotation, SMURF
	Kojic acid	8273	AFLA_096030 - AFLA_096060	4	3	[10], [11]; Blastp, E0.0
	Ustiloxin B	21857	AFLA_094940 – AFLA_095110	18	?	This study
	Cluster 3	7369	AFLA_005320 - AFLA_005350	4	8	SMURF
	Cluster 5	1960	AFLA_006170 - AFLA_006190	3	7	SMURF
	Cluster 7	5193	AFLA_009980 - AFLA_010030	6	8	SMURF
	Cluster 8	9341	AFLA_010600 - AFLA_010630	4	10	SMURF
	Cluster 10	18356	AFLA_023000 – AFLA_023040	5	15	SMURF
	Cluster 17	1423	AFLA_054370 – AFLA_054390	3	25	SMURF
	Cluster 18	1072	AFLA_060030 - AFLA_060050	3	15	SMURF
	Cluster 19	26351	AFLA_060660 - AFLA_060700	5	9	SMURF
	Cluster 20	2079	AFLA_062820 - AFLA_062900	9	18	SMURF
	Cluster 21	8227	AFLA_064260 - AFLA_064330	8	21	SMURF
	Cluster 23	5702	AFLA_066690 – AFLA_066720	4+6	33	SMURF
		2888	AFLA_066890 - AFLA_066940			
	Cluster 24	4508	AFLA_069320 - AFLA_069340	3	10	SMURF
	Cluster 25	4219	AFLA_070860 – AFLA_080890	4+4	26	SMURF
		5148	AFLA_070910 - AFLA_070950			
	Cluster 27	2012	AFLA_082140 - AFLA_082160	3	14	SMURF
	Cluster 33	5797	AFLA_101700 - AFLA_101770	8	6	SMURF
	Cluster 36	1026	AFLA_105410 – AFLA_105450	5	5	SMURF
	Cluster 37	13236	AFLA_108550 – AFLA_108580	4	18	SMURF
	Cluster 41	2503	AFLA_116130 – AFLA_116150	3+3	26	SMURF
		1331	AFLA_116170 – AFLA_116190			
	Cluster 44	6277	AFLA_118390 – AFLA_118410	3	11	SMURF
	Cluster 45	2494	AFLA_118940 – AFLA_119000	7	19	SMURF
	Cluster 46	4420	AFLA_119080 - AFLA_119120	5	6	SMURF
	Cluster 47	12300	AFLA_121470 - AFLA_121540	8	8	SMURF
	Cluster 49	1429	AFLA_128030 - AFLA_128110	9	13	SMURF
	Cluster 53	2813	AFLA_137830 – AFLA_137860	4+3	15	SMURF
		1844	AFLA_137890 – AFLA_137910			

The detection threshold is >95th quantile (false positive rate 0.05).

The most induced combinations of culture conditions are listed in Appendix S2.

aClusters with numbers are those predicted by SMURF. The list of the predicted gene clusters can be downloaded from http://jcvi.org/smurf/precomputed.php.

bGene IDs are for annotated genome sequences in GenBank (A. oryzae, F. verticillioides, and A. flavus) as described in Appendix S1.

cTwo numbers are described when the predicted clusters are divided into two regions and represent the corresponding clusters.

dCluster size experimentally validated or predicted by SMURF (refer to Source in detail).

The cluster harboring fusaric acid biosynthetic genes (peak *c* in Fig. 3B) was predicted to have 17 genes (FVEG_12519−FVEG_12535) by MIDDAS-M, whereas the cluster size reported by Brown et al was 5 (FVEG_12519−FVEG_12523) [23] (Table 1, Fig. S2 in Appendix S1). The gene expression profile in this region suggests existence of another cluster adjacent to the fusaric acid gene cluster with a few additional genes in between (Fig. S2 in Appendix S1). One of the remarkable features of MIDDAS-M is the potential to predict a gene cluster even though it includes a small number of genes that are not co-regulated with other cluster member genes. This enables sensitive detection of gene clusters from the dataset containing inaccurate data points due to their low expression levels and/or biological fluctuation under the same condition. It is thought that this characteristic led to the prediction of the above cluster much longer than the actual size by combining the two clusters into one. In addition to detecting the five clusters noted above, this analysis revealed two other VCs with high ω_max_ scores (*y1* and *y2* in Fig. 3B). They were not predicted by SMURF, and were composed of 3 and 4 genes, respectively, the latter of which included an NRPS-like enzyme (Fig. 3B, Table S2 in Appendix S1). To assign peaks to their corresponding compounds, detailed analysis of the linkage between the gene cluster expression and compound productivity is necessary.

### Large-scale detection of SMB gene clusters by MIDDAS-M

**Figure 4:**
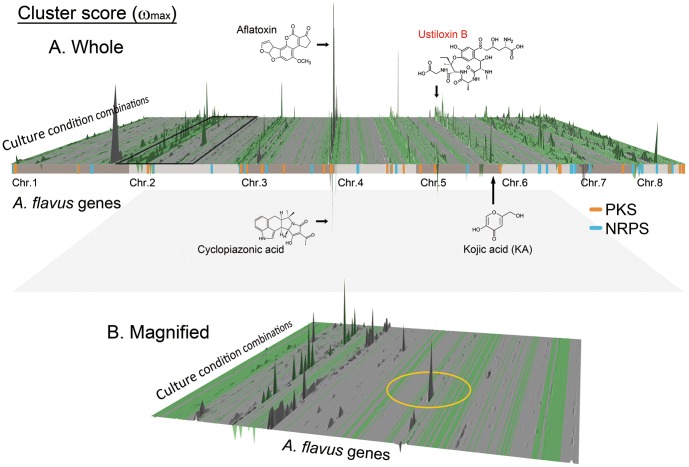
SMB gene cluster detection by MIDDAS-M in *A. flavus*. (A) A 3D view of the ω_max_ scores for all genes and combinations of culture conditions. Comprehensive detection of SMB gene clusters was performed on all 378 pairwise combinations of culture conditions from 28 transcriptomes. The gray and green areas denote blocks of synteny and non-synteny, respectively, with the *A. nidulans* genome. The positions of gene clusters possessing PKS and NRPS core genes predicted by SMURF are shown in orange and blue, respectively. The chemical structures of four *A. flavus* secondary metabolites are shown at the positions of corresponding SMB gene clusters; the ustiloxin B gene cluster was first identified in this paper. (B) Magnified view of the area on chromosome 2 corresponding to the black square in A. As an example, a yellow circle designates the peak observed specifically at particular positions, from which conditions for producing the corresponding compound were determined.

To demonstrate the fully computational and motif-independent features of MIDDAS-M for the comprehensive analysis of SMB gene clusters, we employed a systematic pairwise comparison of *A. flavus* 28 transcriptome datasets from a variety of cultivation conditions (GSE15435 [26], Fig. 4A). MIDDAS-M detected 240 candidate clusters with the threshold of 0.05 for the statistical likelihood of false positives (ω_max_ ≥1,016.7) in a total of 378 pairs of datasets. The results included all 4 experimentally-validated clusters, those for aflatoxin, aflatrem, cyclopiazonic acid, and KA (Table 1). Using the datasets above, twenty-seven of the 55 clusters predicted by SMURF were detected by MIDDAS-M (Table 1). Secondary metabolites tend to be produced under only limited culture conditions; in other words, SMB genes are silent under most conditions. In addition, many SMB-like gene clusters may have possibly lost their functions. For example, *A. oryzae* has the gene cluster homologous to that for aflatoxin in *A. flavus*, but never produces the compound due to mutations both inside and outside the cluster [27]. SMURF, which uses only genome sequence information, predicts clusters regardless of their silence or non-functionality. In contrast, MIDDAS-M excludes non-functional SMB gene clusters in defined culture conditions. Similarly, MIDDAS-M predicted 35 of the 76 candidate clusters predicted by antiSMASH (the column D in the “antiSMASH.AF” sheet in Appendix S2). Certain peaks were detected under only limited combinations of conditions, illustrating the utility of MIDDAS-M for the comprehensive analysis of culture conditions that induce rarely expressed SMB genes (Fig. 4B). For example, the peak circled in Fig. 4B detected only in a limited conditions, composed of AFLA_035680 through AFLA_035720, was not detected either by SMURF or by antiSMASH.

**Figure 5:**
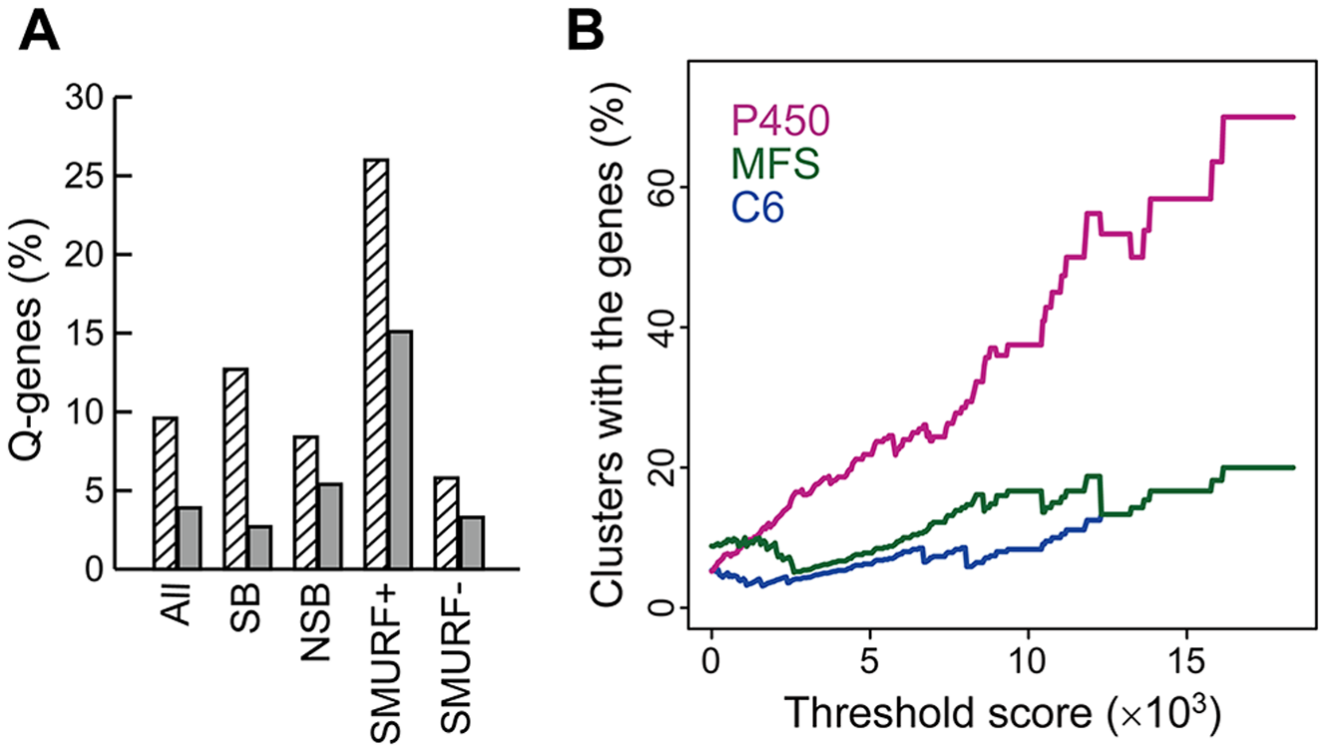
Frequency of SMB-related genes in clusters detected by MIDDAS-M. (A) Ratios of SMB-related genes (Q-genes) detected by KOG analysis with the cluster genes detected by MIDDAS-M (hatched bars) and all the genes in the corresponding genome (gray bars). (B) The proportion of clusters containing genes annotated as P450 enzymes (pink), C6 transcription factors (blue), and major facilitator superfamily members (green) were calculated for detected clusters with the threshold score of ω_max_ in *A. flavus*. The value is plotted to a ω_max_ of 18,350, at which 10 clusters remain to be detected.

The detected peaks were highly localized to NSBs (702 detected cluster genes out of 969 total; see Table S3 in Appendix S1). This result is in good agreement with the fact that the genes related to secondary metabolite biosynthesis, transport, and catabolism (Q-genes), identified in the EuKaryotic Orthologous Groups (KOG) [28], [29] on NSBs [13]. In addition, the detected gene clusters were enriched for Q-genes compared with the whole genome, regardless of their inclusion of core genes (SMURF+/−) (Fig. 5A). Genes annotated as cytochrome P450 enzymes, which constitute a large enzyme family often involved in SMB gene clusters [30], represent 1.1% of the 13,471 genes in the *A. flavus* genome, and are contained in 9.1% of the 240 unique clusters detected by MIDDAS-M. The P450 gene content in the detected gene clusters increased drastically to >60%, by applying threshold ω_max_ ≥15,800 (Fig. 5B), although the number of clusters decreased exponentially along with increasing the threshold of ω_max_ score (24 clusters when ω_max_ ≥10,000, Fig. S3 in Appendix S1). SMB clusters are often regulated by C6-type transcription factors [31], and major facilitator superfamily (MFS) transporters are often present in SMB clusters [32]. These two genes also appear more frequently in the clusters as the threshold increased. Among 240 candidate SMB gene clusters detected by MIDDAS-M with the threshold of 0.05 false positive rate, 89% (213) were not detected by SMURF (Table S3 in Appendix S1), and this tendency continued when ω_max_ >10,000 (71% or 17 in 24). These results strongly suggest that MIDDAS-M detected clusters of SMBs even when the clusters did not include the core genes. Detection of the KA cluster is the typical example. The ustiloxin B biosynthetic gene cluster, which was first detected by MIDDAS-M and experimentally-validated in this study, is another good example. These two clusters are both lacking known core genes, thus have never been predicted by the existing software tools based on sequence information of core genes, such as SMURF and antiSMASH (see detail in the next section). Use of high threshold of ω_max_ and gene functional information will increase accuracy of predicting SMB gene clusters, though it may fail to detect novel SMB clusters.

### Identification of a novel ustiloxin B gene cluster by MIDDAS-M

**Figure 6:**
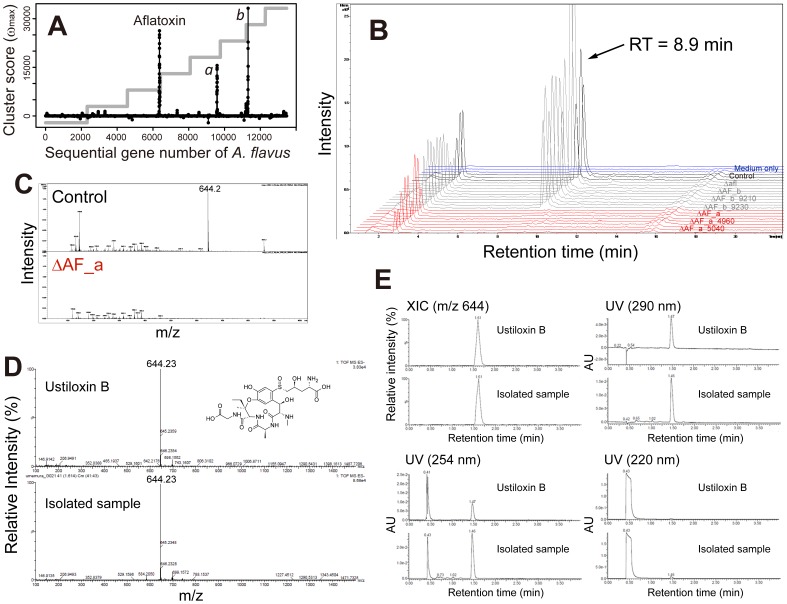
Identification of the ustiloxin B cluster in *A. flavus* based on the MIDDAS-M prediction. (A) MIDDAS-M results from a combination of culture conditions in maize at 28°C versus 37°C. The leftmost distinct peak corresponds to the aflatoxin gene cluster. The other two peaks were designated as clusters *a* and *b*. The step line plot in gray denotes the chromosomes. (B) Peaks at a retention time of 8.9 min detected in the extracted ion chromatograms of m/z 644.2±0.1 in negative ion mode were not observed in the *A. flavus* deletion mutants of the genes in cluster *a* (red). Chromatograms are for medium only (blue, negative control), the control strain (*pyrG* revertant, black), the aflatoxin cluster deletion mutant, and three mutants with deletions in cluster *b* (gray). (C) The mass spectra at of the 8.9 min retention peaks in the control strain (above) and the deletion mutant ΔAF_*a* (below). The MS peak of m/z 644.2 in the control strain was not present in the deletion mutant. (D) Comparison of the mass spectra for ustiloxin B and the compound with m/z 644.2 (in negative ion mode) isolated from the control strain. (E) Comparison of the chromatograms of the ustiloxin B reference standard and the compound isolated in this study. The extracted ion chromatogram of m/z 644.23 in negative ion mode and UV chromatograms at 290, 254, and 220 nm are indicated.

The comprehensive analysis of *A. flavus* transcriptomes by MIDDAS-M revealed a pair of culture conditions (cracked maize at 28°C versus 37°C) that showed 3 distinct peaks: the first peak corresponded to the aflatoxin biosynthetic gene cluster; the second peak to a putative cluster (designated cluster *a*) consisting of 18 genes (AFLA_094940−AFLA_095110; gene ID interval  =  10 in most cases); and the third peak to a putative cluster (cluster *b*) consisting of 5 genes (AFLA_039200−AFLA_039240) (Fig. 6A). To identify the compounds produced by clusters *a* and *b*, we constructed three types of *A. flavus* deletion mutants for each cluster using *pyrG* as a selectable marker. For cluster *a*, mutant ΔAF_*a* had 13 genes (ΔAFLA_094940−AFLA_095060) deleted, mutant ΔAF_*a*_4960 had one gene (ΔAFLA_094960) deleted, and mutant ΔAF_*a*_5040 had one gene (ΔAFLA_095040) deleted. For cluster *b*, mutant ΔAF_*b* had five genes (ΔAFLA_039200−AFLA_039240) deleted, mutant ΔAF_*b*_9210 had one gene (ΔAFLA_039210) deleted, and mutant ΔAF_*b*_9230 had one gene (ΔAFLA_039230) deleted (Fig. S1 and Table S1 in Appendix S1). The deletion mutant lacking the entire aflatoxin cluster and the *pyrG* revertant were also constructed as positive controls. After solid cultivation of the 7 deletion mutants and the control strain (*pyrG* revertant) on cracked maize at 28°C for 7 days, water-soluble metabolites were analyzed by high-performance liquid chromatography-mass spectrometry (HPLC-MS). By comparing metabolite profiles between mutants, we found a negative ion spectrum at m/z 644.2 with a retention time (RT) of 8.9 min that was absent only in water extracts from the three deletion mutants corresponding to cluster *a* (Figs. 6B, 6C). Ultra-performance liquid chromatography-high-resolution mass spectrometry (UPLC-HRMS) showed that the accurate mass of the corresponding ion was 646.240 [M+H]^+^ and 644.231 [M-H]^−^ with UV absorption at 290, 250, and 209 nm. By searching an organic compound database, we found that these measurements corresponded to ustiloxin B (C_26_H_39_N_5_O_12_S; MW 645.681). Ustiloxin B was first isolated as a water-soluble component of false smut balls on rice panicles infected by the fungus *Ustilaginoidea virens* [33]–[35]. The HPLC-purified compound from the water extract of the control strain (*pyrG* revertant) was compared with a ustiloxin B standard using UPLC-HRMS. The two compounds showed identical mass spectra with an RT of 1.61 min (Fig. 6D) as well as identical peaks in the extracted ion chromatogram at m/z 644.231 [M-H]^−^ and in the UV spectra at 290, 254, and 220 nm (Fig. 6E). These results provide the first evidence that the genes AFLA_094960 and AFLA_095040 are responsible for ustiloxin B biosynthesis, indicating that cluster *a*, composed of AFLA_094940 through AFLA_095110, is a ustiloxin B biosynthetic cluster.

Based on its chemical structure, ustiloxin B is likely characterized as a non-ribosomal peptide. One of the genes responsible for producing ustiloxin B, AFLA_095040, was putatively annotated as an NRPS-like enzyme in the NCBI database (gene ID: 7917917). However, the AFLA_095040 gene contains only the catalytic domain of a pyridoxal 5′-phosphate-dependent enzyme from aminotransferase family-5, which must be involved in reactions other than non-ribosomal peptide bond biosynthesis (Fig. S4 in Appendix S1 and the “ust” sheet in Appendix S2). Moreover, none of the NRPS-specific catalytic domains (A, C, PCP, or TE) were found in any genes in or near the cluster (AFLA_094930−AFLA_095170), as determined by a BLAST [36] search against the UniProtKB database [37], [38]. Accordingly, the cluster was not detected by SMURF (http://jcvi.org/smurf/precomputed.php), antiSMASH (the “antiSMASH.AF” sheet in Appendix S2), or other currently available conventional SMB gene cluster prediction methods, which use catalytic domain sequence motif information. This result clearly indicates that MIDDAS-M has potential use as a motif-independent predictor of functional SMB gene clusters.

## Discussion

In this work, we described the first sequence motif-independent algorithm for the discovery of functional fungal SMB gene clusters based on a combination of whole genome sequence data and transcriptome information. To achieve this novel and fully computational approach, we combined an algorithm to generate comprehensive virtual gene clusters on a genome of interest with the statistical processing of signal enhancement based on deviation from a standard distribution for transcriptional induction or repression of a cluster. First, we confirmed that our algorithm, MIDDAS-M, accurately detected experimentally validated SMB gene clusters, including the fumonisin, aflatoxin/sterigmatocystin, and KA clusters, from DNA microarray datasets obtained under culture conditions associated with the production and non-production of these compounds. In contrast to the former 3 clusters, the KA gene cluster does not include any genes considered as core SMB genes, such as PKSs, NRPSs, DMATs, or terpene cyclases (TCs). The KA gene cluster predicted by MIDDAS-M was the sole candidate with a correct cluster size. Nine gene disruption experiments were required to identify this cluster without MIDDAS-M prediction in our previous work using the same transcriptomes [11].

The fully computational and motif-independent feature of MIDDAS-M allowed for the comprehensive analysis of SMB gene clusters based on expression differences in a given pair of multiple transcriptomes. Because little is known about SMB gene clusters other than those containing PKS, NRPS, TC, and DMATS, the validation of the MIDDAS-M results is extremely difficult. Nonetheless, based on the MIDDAS-M prediction, we identified the first SMB gene cluster for ustiloxin B, the non-ribosomal peptide-like compound that inhibits microtubule assembly [35], in *A. flavus*. Although ustiloxin B was identified more than 20 years ago, the ustiloxin B biosynthetic gene cluster had remained unknown until the present study. The lack of the NRPS catalytic domains A, C, PCP, and TE in all genes both in the cluster and within 10 adjacent genes outside the cluster strongly suggests a novel mechanism for cyclic peptide biosynthesis. Our further deletion experiments and sequence analysis revealed that at least 3 genes with unknown functions (AFLA_094970, AFLA_094980, and AFLA_094990) may be involved in the peptide bond synthesis and cyclization of the compound, supporting the idea above (data not shown). However, there still remains a possibility that additional gene encoding an NRPS for the ustiloxin biosynthesis may be located distantly from the cluster.

MIDDAS-M enables the highly sensitive identification of SMB gene clusters, but the predicted cluster sizes may be smaller than the actual cluster sizes in some cases. For example, the aflatoxin gene cluster of *A. flavus* is composed of 29 genes from AFLA_139150 through AFLA_139440 [39], [40], but MIDDAS-M detected 23 genes, AFLA_139150 through AFLA_139410 (excluding AFLA_139330 – AFLA_139360). This discrepancy is most likely due to the *Z*-score transformation at each *ncl* used to normalize *M* scores before enhancement. When information from a candidate gene cluster(s) is included at a certain *ncl*, the standard deviation used for the denominator in *Z*-score transformation increases. As a result, the *M* score(s) of the strongly positive gene cluster tend to be smaller at the correct size. This factor does not affect the detection sensitivity of cluster positions but does affect the cluster boundary detection. One potential solution for this problem is to use another algorithm, such as co-expression analysis, for the precise prediction of cluster boundaries after the sensitive detection of cluster candidates by MIDDAS-M.

There are more than 100,000 fungal species in nature [41] that are potential producers of bioactive compounds [31]. Because fungal SMB genes are highly divergent [16], [42], [43], even fungal species closely related to those that have already been sequenced are worth sequencing to discover new SMB genes. We have confirmed that MIDDAS-M performs equally well when using transcriptomes from RNA-seq data in a comparative performance with DNA microarray for SMB gene cluster detection. MIDDAS-M enables the comprehensive exploration of functional SMB genes in fungal genomes by effectively utilizing the vast amount of available genome and transcriptome information, which will accelerate the discovery of biosynthesis or other functional categories of genes in the future.

## Supporting Information

Appendix S1 Experimental details pertaining to the algorithm execution using transcriptome data, gene disruption, and the identification of ustiloxin B.
(DOCX)

Appendix S2 The comprehensive MIDDAS-M prediction data for *F. verticillioides* (the “F.verticillioides” sheet) and *A. flavus* (the “A.flavus” sheet), the functional annotations of genes in or near the ustiloxin B cluster found by BLAST against UniProtKB (the “ust” sheet), and the result of antiSMASH prediciton for *A. flavus* in comparison to that of MIDDAS-M (the “antiSMASH.AF” sheet).
(XLSX)

## References

[1] Yu J, Bhatnagar D, Ehrlich KC (2002) Aflatoxin biosynthesis. Rev Iberoam Micol 19: 191–200. 12825981

[2] Rank C, Larsen TO, Frisvad JC (2010) Functional systems biology of Aspergillus. In: Machida M, Gomi K, editors. *Aspergillus* Molecular Biology and Genomics. Norfolk, UK: Caister Academic Press. pp. 173–198.

[3] Khaldi N, Seifuddin FT, Turner G, Haft D, Nierman WC, et al (2010) SMURF: Genomic mapping of fungal secondary metabolite clusters. Fungal Genet Biol 47: 736–741. 20554054 10.1016/j.fgb.2010.06.003 PMC2916752

[4] Medema MH, Blin K, Cimermancic P, de Jager V, Zakrzewski P, et al (2011) antiSMASH: rapid identification, annotation and analysis of secondary metabolite biosynthesis gene clusters in bacterial and fungal genome sequences. Nucleic Acids Res 39: W339–346. 21672958 10.1093/nar/gkr466 PMC3125804

[5] Blin K, Medema MH, Kazempour D, Fischbach MA, Breitling R, et al (2013) antiSMASH 2.0 − a versatile platform for genome mining of secondary metabolite producers. Nucleic Acids Res 41: W204–W212. 23737449 10.1093/nar/gkt449 PMC3692088

[6] Weber T, Rausch C, Lopez P, Hoof I, Gaykova V, et al (2009) CLUSEAN: a computer-based framework for the automated analysis of bacterial secondary metabolite biosynthetic gene clusters. J Biotechnol 140: 13–17. 19297688 10.1016/j.jbiotec.2009.01.007

[7] Andersen MR, Nielsen JB, Klitgaard A, Petersen LM, Zachariasen M, et al (2012) Accurate prediction of secondary metabolite gene clusters in filamentous fungi. Proc Natl Acad Sci U S A 110: E99–107. 23248299 10.1073/pnas.1205532110 PMC3538241

[8] Chang PK, Horn BW, Dorner JW (2005) Sequence breakpoints in the aflatoxin biosynthesis gene cluster and flanking regions in nonaflatoxigenic *Aspergillus flavus* isolates. Fungal Genet Biol 42: 914–923. 16154781 10.1016/j.fgb.2005.07.004

[9] Brodhun F, Feussner I (2011) Oxylipins in fungi. FEBS J 278: 1047–1063. 21281447 10.1111/j.1742-4658.2011.08027.x

[10] Marui J, Yamane N, Ohashi-Kunihiro S, Ando T, Terabayashi Y, et al (2011) Kojic acid biosynthesis in *Aspergillus oryzae* is regulated by a Zn(II)(2)Cys(6) transcriptional activator and induced by kojic acid at the transcriptional level. J Biosci Bioeng 112: 40–43. 21514215 10.1016/j.jbiosc.2011.03.010

[11] Terabayashi Y, Sano M, Yamane N, Marui J, Tamano K, et al (2010) Identification and characterization of genes responsible for biosynthesis of kojic acid, an industrially important compound from *Aspergillus oryzae* . Fungal Genet Biol 47: 953–961. 20849972 10.1016/j.fgb.2010.08.014

[12] Saito K (1907) Uber die Saurebinding von *Aspergillus oryzae* . Botanical Magazine Tokyo 21: 7–11.

[13] Machida M, Asai K, Sano M, Tanaka T, Kumagai T, et al (2005) Genome sequencing and analysis of *Aspergillus oryzae* . Nature 438: 1157–1161. 16372010 10.1038/nature04300

[14] Galagan JE, Calvo SE, Cuomo C, Ma LJ, Wortman JR, et al (2005) Sequencing of *Aspergillus nidulans* and comparative analysis with *A. fumigatus* and *A. oryzae* . Nature 438: 1105–1115. 16372000 10.1038/nature04341

[15] Nierman WC, Pain A, Anderson MJ, Wortman JR, Kim HS, et al (2005) Genomic sequence of the pathogenic and allergenic filamentous fungus *Aspergillus fumigatus* . Nature 438: 1151–1156. 16372009 10.1038/nature04332

[16] Machida M, Terabayashi Y, Sano M, Yamane N, Tamano K, et al (2008) Genomics of industrial *Aspergilli* and comparison with toxigenic relatives. Food Addit Contam Part A Chem Anal Control Expo Risk Assess 25: 1147–1151. 18798040 10.1080/02652030802273114

[17] Tamano K, Sano M, Yamane N, Terabayashi Y, Toda T, et al (2008) Transcriptional regulation of genes on the non-syntenic blocks of *Aspergillus oryzae* and its functional relationship to solid-state cultivation. Fungal Genet Biol 45: 139–151. 17967552 10.1016/j.fgb.2007.09.005

[18] Umemura M, Koike H, Yamane N, Koyama Y, Satou Y, et al (2012) Comparative genome analysis between *Aspergillus oryzae* strains reveals close relationship between sites of mutation localization and regions of highly divergent genes among *Aspergillus* species. DNA Res 19: 375–382. 22912434 10.1093/dnares/dss019 PMC3473370

[19] Cabanes J, Chazarra S, Garcia-Carmona F (1994) Kojic acid, a cosmetic skin whitening agent, is a slow-binding inhibitor of catecholase activity of tyrosinase. J Pharm Pharmacol 46: 982–985. 7714722 10.1111/j.2042-7158.1994.tb03253.x

[20] Bentley R (2006) From miso, sake and shoyu to cosmetics: a century of science for kojic acid. Nat Prod Rep 23: 1046–1062. 17119644 10.1039/b603758p

[21] Futamura T, Ishihara H, Tamura T, Yasutake T, Huang G, et al (2001) Kojic acid production in an airlift bioreactor using partially hydrolyzed raw corn starch. J Biosci Bioeng 92: 360–365. 16233111 10.1263/jbb.92.360

[22] Wan HM, Chen CC, Giridhar R, Chang TS, Wu WT (2005) Repeated-batch production of kojic acid in a cell-retention fermenter using *Aspergillus oryzae* M3B9. J Ind Microbiol Biotechnol 32: 227–233. 15895266 10.1007/s10295-005-0230-5

[23] Brown DW, Butchko RA, Busman M, Proctor RH (2012) Identification of gene clusters associated with fusaric acid, fusarin, and perithecial pigment production in *Fusarium verticillioides* . Fungal Genet Biol 49: 521–532. 22652150 10.1016/j.fgb.2012.05.010

[24] Proctor RH, Brown DW, Plattner RD, Desjardins AE (2003) Co-expression of 15 contiguous genes delineates a fumonisin biosynthetic gene cluster in *Gibberella moniliformis* . Fungal Genet Biol 38: 237–249. 12620260 10.1016/s1087-1845(02)00525-x

[25] Wiemann P, Willmann A, Straeten M, Kleigrewe K, Beyer M, et al (2009) Biosynthesis of the red pigment bikaverin in *Fusarium fujikuroi*: genes, their function and regulation. Mol Microbiol 72: 931–946. 19400779 10.1111/j.1365-2958.2009.06695.x

[26] Georgianna DR, Fedorova ND, Burroughs JL, Dolezal AL, Bok JW, et al (2010) Beyond aflatoxin: four distinct expression patterns and functional roles associated with *Aspergillus flavus* secondary metabolism gene clusters. Molecular Plant Pathology 11: 213–226. 20447271 10.1111/j.1364-3703.2009.00594.x PMC4116135

[27] Kusumoto K, Nogata Y, Ohta H (2000) Directed deletions in the aflatoxin biosynthesis gene homolog cluster of *Aspergillus oryzae* . Curr Genet 37: 104–111. 10743566 10.1007/s002940050016

[28] Tatusov RL, Fedorova ND, Jackson JD, Jacobs AR, Kiryutin B, et al (2003) The COG database: an updated version includes eukaryotes. BMC Bioinformatics 4: 41. 12969510 10.1186/1471-2105-4-41 PMC222959

[29] Tatusov RL, Koonin EV, Lipman DJ (1997) A genomic perspective on protein families. Science 278: 631–637. 9381173 10.1126/science.278.5338.631

[30] Weitzel C, Simonsen HT (2013) Cytochrome P450-enzymes involved in the biosynthesis of mono- and sesquiterpenes. Phytochemistry Reviews.

[31] Keller NP, Turner G, Bennett JW (2005) Fungal secondary metabolism - from biochemistry to genomics. Nat Rev Microbiol 3: 937–947. 16322742 10.1038/nrmicro1286

[32] Coleman JJ, Mylonakis E (2009) Efflux in fungi: la piece de resistance. PLoS Pathog 5: e1000486. 19557154 10.1371/journal.ppat.1000486 PMC2695561

[33] Koiso Y, Li Y, Iwasaki S, Hanaoka K, Kobayashi T, et al (1994) Ustiloxins, antimitotic cyclic peptides from false smut balls on rice panicles caused by *Ustilaginoidea virens* . J Antibiot (Tokyo) 47: 765–773. 8071121 10.7164/antibiotics.47.765

[34] Koiso Y, Natori M, Iwasaki S, Sato S, Sonoda R, et al (1992) Ustiloxin: A phytotoxin and a mycotoxin from false smut balls on rice panicles. Tetrahedron Letters 33: 4157–4160.

[35] Koiso Y, Morisaki N, Yamashita Y, Mitsui Y, Shirai R, et al (1998) Isolation and structure of an antimitotic cyclic peptide, ustiloxin F: chemical interrelation with a homologous peptide, ustiloxin B. J Antibiot (Tokyo). 51: 418–422. 10.7164/antibiotics.51.418 9630863

[36] Altschul SF, Gish W, Miller W, Myers EW, Lipman DJ (1990) Basic local alignment search tool. J Mol Biol 215: 403–410. 2231712 10.1016/S0022-2836(05)80360-2

[37] Punta M, Coggill PC, Eberhardt RY, Mistry J, Tate J, et al (2012) The Pfam protein families database. Nucleic Acids Res 40: D290–301. 22127870 10.1093/nar/gkr1065 PMC3245129

[38] Consortium U (2011) Reorganizing the protein space at the Universal Protein Resource (UniProt). Nucleic Acids Res 40: D71–75. 22102590 10.1093/nar/gkr981 PMC3245120

[39] Georgianna DR, Payne GA (2009) Genetic regulation of aflatoxin biosynthesis: from gene to genome. Fungal Genet Biol 46: 113–125. 19010433 10.1016/j.fgb.2008.10.011

[40] Yu J (2012) Current understanding on aflatoxin biosynthesis and future perspective in reducing aflatoxin contamination. Toxins (Basel) 4: 1024–1057. 23202305 10.3390/toxins4111024 PMC3509697

[41] Hawksworth DL (2004) Fungal diversity and its implications for genetic resource collections. Studies in Mycology 50: 9–18.

[42] Sanchez JF, Somoza AD, Keller NP, Wang CC (2012) Advances in *Aspergillus* secondary metabolite research in the post-genomic era. Nat Prod Rep 29: 351–371. 22228366 10.1039/c2np00084a PMC4568942

[43] Yin W, Keller NP (2011) Transcriptional regulatory elements in fungal secondary metabolism. J Microbiol 49: 329–339. 21717315 10.1007/s12275-011-1009-1 PMC3714018

[44] Nicholson MJ, Koulman A, Monahan BJ, Pritchard BL, Payne GA, et al (2009) Identification of two aflatrem biosynthesis gene loci in *Aspergillus flavus* and metabolic engineering of *Penicillium paxilli* to elucidate their function. Appl Environ Microbiol 75: 7469–7481. 19801473 10.1128/AEM.02146-08 PMC2786402

[45] Chang PK, Horn BW, Dorner JW (2009) Clustered genes involved in cyclopiazonic acid production are next to the aflatoxin biosynthesis gene cluster in *Aspergillus flavus* . Fungal Genet Biol 46: 176–182. 19038354 10.1016/j.fgb.2008.11.002

