# Supplementary material for: MIDDAS-M: Motif-Independent De Novo Detection of Secondary Metabolite Gene Clusters through the Integration of Genome Sequencing and Transcriptome Data
Source: PLoS One. 2013 Dec 31;8(12):e84028. doi: 10.1371/journal.pone.0084028 (PMC3877130; doi:10.1371/journal.pone.0084028)
Supplement: Appendix S1 — Experimental details pertaining to the algorithm execution using transcriptome data, gene disruption, and the identification of ustiloxin B. (DOCX) [file pone.0084028.s001.docx]

**Supplementary Methods**

**Genome sequences.** We used the following annotated genome sequences to create virtual gene clusters (VCs) in MIDDAS-M: GenBank CM000578-CM000588 from the Fusarium Comparative Sequencing Project at the Broad Institute of Harvard and MIT (http://www.broadinstitute.org) for *Fusarium verticillioides* [1], GenBank AP007150-AP007177 for *Aspergillus oryzae* [2], and GenBank EQ963472-EQ966232 for *Aspergillus flavus*. Homology searches for *F. verticillioides* genes were performed with BLASTp of the nonredundant database of the National Center for Biotechnology Information (NCBI-NR) [3] for gene annotations; the top hit sequence was used to annotate the query gene.

**Transcriptome data for MIDDAS-M calculation.** The transcriptome datasets used were isolated from *A. oryzae* under kojic acid production conditions (accession no. GSE43280 [4]), from *F. verticillioides* under a time course of fumonisin production conditions (accession no. GSE16900 [5]), and from *A. flavus* under 28 cultivation and mutation conditions tested to investigate the mechanisms of secondary metabolite production (accession no. GSE15435 [5]), all of which can be downloaded from the Gene Expression Omnibus database of the NCBI [6,7] (http://www.ncbi.nlm.nih.gov/geo/).

The *A. oryzae* transcriptome data reflected the relative abundances of transcripts compared between KA-producing and KA-non-producing conditions obtained from two-color microarray experiments. The transcriptome data for *F. verticillioides* and *A. flavus* were obtained in the form of absolute expression levels, and the comprehensive pairwise comparison was performed using their logarithmic values after averaging over replications for the MIDDAS-M computation.

**MIDDAS-M computation.** Transcriptome data such as induction ratios obtained from 2-color DNA microarray experiments may be used directly for MIDDAS-M analysis. When the data are provided as absolute expression values, such as those of 1-color DNA microarray and RNA-seq, MIDDAS-M generates all possible pairwise combinations (not permutations) of transcriptome datasets, followed by the subtraction of the datasets in logarithm form to evaluate induction ratios for each gene.

In the preparation of virtual gene clusters (VCs) from previously gene-annotated genome sequences, all gene clusters of sizes (*ncl*s) from 3 to an appropriate upper limit (30 in this study) were determined; the clusters at the margin of a scaffold or chromosome were not included unless they met the cluster size. Any genes that were not found in a transcriptome were assigned a value of zero. In evaluating the ω score, the probability of each *M* score at each *ncl* was evaluated from the histogram of all *M* scores divided into 100 segments. Among the clusters beginning from the same gene with *ncl* of 3 to 30, the cluster showing the largest absolute value of ω (ω_max_) was assigned to the gene (the “Maximum score” column in Supplementary Data, “F.verticillioides” and “A.flavus” sheets). Finally, the set of unique, non-overlapping clusters was defined (the “Unique cluster” column in Supplementary Data, “F.verticillioides” and “A.flavus” sheets). For example, assume 5 contiguous genes with ω_max_ of 1, 4, 10, 8, and 5, and *ncl* of 5, 4, 3, 2, and 1. In this case, the unique cluster is composed of the 3rd to 5th genes because the cluster showing the highest score starts at the 3^rd^ gene and has an *ncl* of 3. All genes in a unique cluster have the same ω_max_ and *ncl*.

The threshold score was set to a 0.05 false-positive probability for the ω_max_ of unique clusters, which was the 95% quantile value of ω_max_ deduced from the same dataset with a random gene order. The calculated threshold scores were as follows: *F. verticillioides*, 499.4; *A. flavus*, 1,016.7.

Codes for the calculation of *M* (Eq.1) and ω (Eq.2) were written in Perl and R [8], respectively, and executed on a Linux Real Computing RX2000 server with Scientific Linux 5.5. The required memory space was approximately 20 gigabytes, and ~20 min with a single CPU of AMD Opteron 2.2 GHz was needed to complete the calculation for 28 *A. flavus* transcriptomes including 13,471 genes. MIDDAS-M is available for use at the following server (http://133.242.13.217/MIDDAS-M).

**Analysis of the SMB cluster candidates detected by MIDDAS-M.** The syntenic blocks (SBs) in the *A. flavus* genome were defined against the *A. nidulans* genome according to the identification of orthologs followed by the identification of conserved contiguous blocks within a window of 10 kb, as described previously [2]. Regions outside SBs were defined as non-syntenic blocks (NSBs). The functional categories of *A. flavus* genes were assigned according to the eukaryotic orthologous groups (KOG) classification [9,10] by searching for homology against amino acid sequences in the KOG database with a bit score of ≥60, followed by evaluating the occupancy frequency of genes belonging to category Q (secondary metabolite biosynthesis, transport, and catabolism), in the clusters detected by MIDDAS-M. These analyses were performed using BioPerl and R. For comparison, AntiSMASH 2.0.2 was performed for *A. flavus* using the above GenBank files on a DELL precision T7500 desktop computer (CPU, Xeon E5620×2; Memory, 96 GB; harddisk, 2TB×5; OS, Ubuntu Linux 10.04); we also evaluated the results of a SMURF analysis, which can be downloaded from http://jcvi.org/smurf/precomputed.php.

**Strain and media.** *Aspergillus flavus* strain CA14 ∆*ku70* ∆*pyrG* ∆*niaD* was used for the construction of the deletion mutants and the *pyrG* revertant. For DNA isolation, the fungus was grown in liquid YPD (yeast extract, peptone, dextrose) medium (Difco) supplemented with 1.12 g/L of uracil at 30°C for 2 days.

**DNA preparation.** Genomic DNA was isolated from *Aspergillus flavus* strain CA-14 ∆*ku70* ∆*pyrG* ∆*niaD* by first grinding the mycelia to a fine powder in liquid nitrogen, and then mixing ~40 g of the ground mycelia with 200 mL of 50 mM ethylenediaminetetraacetic acid (EDTA), 0.5% SDS, and 0.1 mg/mL Proteinase K (TaKaRa) at pH 8.0 and 50°C. The mixture was incubated for 3 h at 50°C, followed by centrifugation at 2,300 × g for 10 min. The resulting supernatant was extracted with phenol, phenol/chloroform, and chloroform, and the DNA was precipitated using ethanol. The precipitated DNA was dissolved in Tris-EDTA buffer, and the genomic DNA was then purified using a Genomic-tip column (Qiagen).

**Gene disruption and transformation.** The disruption of *A. flavus* genes corresponding to the predicted two SMB gene clusters by MIDDAS-M (*a*, AFLA_094940-AFLA_095060; *b*, AFLA_039200-AFLA_039240) was accomplished via protoplast transformation [11,12] with *pyrG* as the selectable marker [13]. Deletion cassettes were constructed via fusion PCR [14]. Approximately 1 kb of the upstream and downstream regions of each target gene were amplified by the primer pairs of 5F/5R and 3F/3R, respectively (Table S1), using the KOD Plus enzyme (Toyobo) and genomic DNA from *A. flavus* CA-14 ∆*ku70* ∆*pyrG* ∆*niaD* as a template. A *pyrG* fragment originating in *A. nidulans* and flanked by its own promoter and terminator was also amplified with the primer pair pyrG-F/pyrG-R (Table S1). The amplified products were purified using a Wizard SV gene and PCR Clean-Up system kit (Promega), and the second PCR was performed using approximately 10 ng of each purified 5’- and 3’-arm and approximately 40 ng of the *pyrG* fragment with the KOD Plus enzyme. The *A. flavus* *pyrG* gene fragment was also amplified by the primer pair F/R (Table S1) using genomic DNA as a template and purified to construct the *pyrG* revertant.

For fungal transformation, we combined the methods previously published for *A. oryzae* [4] and *A. flavus* [15]. Conidia (10^6^ per gram) of *A. flavus* CA14 ∆*ku70* ∆*pyrG* ∆*niaD* were placed into 300-mL flasks containing 100 mL of potato dextrose broth (Difco) augmented with 1.12 g/L of uracil and incubated at 30°C on a rotary shaker at 170 rpm for two days. The cultures were harvested and washed with 0.8 M NaCl solution. A solution containing 100 mg of lysing enzyme (Sigma), 100 mg of Yatalase (TaKaRa), and 50 mg of cellulase (Yakult) in 30 mL 1 M NaH_2_PO_4_ and 2.5 M NaCl was added to the fungal tissue. This mixture was gently shaken at 100 rpm and 30°C for 3 h. Cell wall debris was removed with a cell strainer (BD Falcon), and the filtrate was centrifuged at 3,500 rpm in an AR510-04 rotor (TOMY) at 4°C for 20 min. After discarding the supernatant, the pellet was washed twice and diluted with 100 μL each of 1.2 M sorbitol, 50 mM CaCl_2_, and 10 mM Trizma base (pH 7.5). Approximately 1 μg of each final DNA fragment was mixed with a 100-μL aliquot of the protoplasts on ice. After incubation on ice for 20 min, 1 mL of 50% polyethylene glycol (M_r_ 3350, Sigma), 1 mL 1 M Tris-HCl (pH 7.5), and 1 mL 1 M CaCl_2_ in a final volume of 100 mL was added, mixed by tapping, and incubated at room temperature for 20 min. Each transformation solution was plated on the surface of regeneration medium (35 g Czapek-Dox broth (Difco), 52.86 g of (NH_4_)_2_SO_4_ (Nacalai Tesque), 10 g agar in a final volume of 1 L). The plates were incubated at 37°C for 3-5 days.

Three putative transformants for each deletion mutant (except ΔAF_*b*_9230 for which only a single transformant could be obtained) were isolated from single conidia, subjected to DNA isolation, and screened by amplifying loci outside and inside the target genes by PCR using the AmpliTaq Gold 360 Master Mix (Applied Biosystems) or KOD FX Neo (Toyobo). The primer pairs cF/cR were constructed for each target gene to amplify the region outside the target genes (Table S1). For cases in which the size of the deleted gene was similar to that of *pyrG*, the primer pairs incF/incR, which amplify regions inside the deleted genes, were also used to check for the absence of the target genes (Table S1). The amplicon sizes show that all deletion mutants and the revertant were successfully obtained. The sizes of the PCR products amplified by the cF/cR primer pairs were approximately 2 kb in all deletion clones and corresponded to the sizes of the target genes in the parent strain, CA-14 ∆*ku70* ∆*pyrG* ∆*niaD*. The whole-gene cluster regions of *a* and aflatoxin could not be amplified even using KOD FX Neo, which can amplify large regions, because the amplicon sizes were 19 and 62 kb, respectively (Figure S1A). In Δ*pyrG*+*pyrG* clones, the amplicon size by cF/cR was approximately 0.8 kb, whereas it was absent in the parent strain, indicating that *pyrG* was successfully restored in the transformants. The regions inside the target genes were not amplified by the incF/incR primer pairs from the deletion mutants but were amplified from the parent strain (Figure S1B), indicating that the genes were deleted as intended.

**Solid medium cultivation and metabolite analysis.** After precultivating 10^6^ conidia in 10 mL of potato dextrose broth (Difco) at 170 rpm at 30°C for 24 h, three clones for each mutant, ΔAF_*a*, ΔAF_*a*_4960, ΔAF_*a*_5040, ΔAF_*b*, ΔAF_*b*_9210, Δ*afl*, and the control strain (*pyrG* revertant), and one clone for ΔAF_*b*_9230, were cultivated at 28°C for 7 days in 50-mL glass vials containing an autoclaved medium consisting of 2.5 g cracked maize and 1.2 mL sterile water. The fungal cultures were then homogenized and extracted with 10 mL of 70% aqueous acetone for 2 h at room temperature. After vaporizing the acetone, 300 μL of the aqueous concentrate was mixed with an equal volume of ethyl acetate at room temperature for 1 h, and the water layers were filtered using filter units with a 0.22 μm pore size (Nacalai Tesque). To compare metabolite profiles, a 2-μL aliquot of each water extract was separated on an Ultimate 3000 HPLC (Dionex) using a 2.0 × 250 mm Develosil XG-C18M-5 reversed-phase column (Nomura Chemical) and eluted with a gradient of water−acetonitrile (100:0 to 0:100 in 30 min) at a flow rate of 0.2 mL/min. A micrOTOF II KIK2 MS (Bruker Daltonics) was used for detection. To isolate the compound with an m/z of 644.2 (in negative ion mode), which was absent in the culture medium of mutants corresponding to *A. flavus* SMB cluster *a* detected by MIDDAS-M (∆AF_*a*, ∆AF_*a*_4960, and ∆AF_*a*_5040), the *pyrG* revertant was cultured in a medium containing 5 g of autoclaved cracked maize and 2.4 mL sterile water at 30°C for 7 days. The compound in 3 mL of the water extract, obtained by the same procedure as described above, was isolated twice on the same HPLC with MS monitoring using a 4.6×250 mm Develosil XG-C18M-5 reversed-phase column (Nomura Chemical) with an isocratic system of 1% aqueous acetonitrile at a flow rate of 1 mL/min. The isolated compound and a ustiloxin B standard were then separated by UPLC-HRMS LCT Premier XE (Waters) using a 2.1×100 mm Acquity UPLC BEH C18 reversed-phase column (Waters) with an isocratic system of 1% aqueous acetonitrile containing 0.1% formic acid at a flow rate of 0.6 mL/min, and their chromatograms were compared.

**

**

**Figure S1. Electrophoresis analysis of PCR products amplified using primer pairs targeting the regions outside and inside deleted genes.** (A) Loci outside the target genes, amplified using the cF/cR primer sets. The DNA polymerase used for ΔAF_*a*, ΔAF_*b*, and Δ*afl* was KOD FX Neo, whereas AmpliTaq Gold 360 was used for ΔAF_*a*_4960, ΔAF_*a*_5040, ΔAF_*b*_9210, and ΔAF_*b*_9230. (B) Loci inside the target genes were amplified using incF/incR primer sets. Lanes: M, 1 kb DNA ladder marker; C, genomic DNA of CA14 ∆*ku70* ∆*pyrG* ∆*niaD* used as a template for each primer set; 1-3, the genomic DNA of each mutant clone used as a template. A 1% agarose gel was used. (C) The diagram showing the positions of the primers, cF/cR and incF/incR, in relation to the structures of wild-type and deleted genes.

**Table S1. *A. flavus* mutants and primers used for the MIDDAS-M experimental validation.**

| Disruptant | Target gene | Primer type | Amplicon size with cF/cR and incF/incR in recipient | Sequence^1^ |
| --- | --- | --- | --- | --- |
| ∆AF_*a* | AFLA_094940 − AFLA_095060 | 5F |  | GGCGGGAGATGTTTGATAATA |
|  |  | 5R |  | gtcagcggccgcatccctgc GGGTCCCGAGTCCTGATAAATATAA |
|  |  | 3F |  | cacggcgcgcctagcagcgg AGTAATCTGTAGATTAGGGCTTTAG |
|  |  | 3R |  | TGTTGTGAGCTTTTGTAAGTGG |
|  |  | cF | 19217 | TATTTATCAGGACTCGGG |
|  |  | cR |  | GCAACAGTATCGACCATA |
| ∆AF_*a*_4960 | AFLA_094960 | 5F |  | GACCATGCTACAAAAATCTCAC |
|  |  | 5R |  | gtcagcggccgcatccctgc TCCTGCTCGGGGCTTCCGTGTGTAT |
|  |  | 3F |  | cacggcgcgcctagcagcgg TGGATTCCAAGGGCTGATGTATTAA |
|  |  | 3R |  | TAATACTCTCTACTGGTGCTGC |
|  |  | cF | 1918 | AGTCAATACACACGGAAG |
|  |  | cR |  | CATCAGCCCTTGGAATCC |
|  |  | incF | 1841 | CATTGACCTTCGCCATCTTA |
|  |  | incR |  | TGCCCTGAAAAGATCCATAT |
| ∆AF_*a*_5040 | AFLA_095040 | 5F |  | TGTGAATGTGTAGTAAGGCAGT |
|  |  | 5R |  | gtcagcggccgcatccctgc TGTCGATGATCCACTTTACTGTGTT |
|  |  | 3F |  | cacggcgcgcctagcagcgg AACTACTCCTCGCTTCCTCTACTCA |
|  |  | 3R |  | GCTCACCATTAATCCACTCATA |
|  |  | cF | 1492 | CAGTAAAGTGGATCATCG |
|  |  | cR |  | AGGAAGCGAGGAGTAGTT |
|  |  | incF | 1259 | GACGGTTGTTCTGAAGGAAG |
|  |  | incR |  | CGTGACAATCTCATCCAACT |
| ∆AF_*b* | AFLA_039200 − AFLA_039240 | 5F |  | CATGCCTTTGTTAGTTATCGTC |
|  |  | 5R |  | gtcagcggccgcatccctgcCTCGCACGCACCTCGTGCTGCCCTA |
|  |  | 3F |  | cacggcgcgcctagcagcggCATGTGCCGAGTCAGCTGTCAATGT |
|  |  | 3R |  | CCCAGCAAATAGACAAGATTAC |
|  |  | cF | 9444 | AGGATAGGATTAGGGCAGCA |
|  |  | cR |  | CCGCTTTAAAGTCAACATTG |
| ∆AF_*b*_9210 | AFLA_039210 | 5F |  | GAAGGGTGAGAGGCAATATTTGA |
|  |  | 5R |  | gtcagcggccgcatccctgc CTATTTTCTTTAACCATTCCGCAAG |
|  |  | 3F |  | cacggcgcgcctagcagcgg TCCGACATAATAGAGCAGGCCTGTA |
|  |  | 3R |  | TATAGAGTGTGTTTGGTGCTGT |
|  |  | cF | 1413 | AGTTGTGATGACCTTGCGGA |
|  |  | cR |  | TACAGGCCTGCTCTATTATG |
|  |  | incF | 1340 | TGAGGGCGCCTACACTACACTC |
|  |  | incR |  | GCGCCTTCTCCGTTTAAATA |
| ∆AF_*b*_9230 | AFLA_039230 | 5F |  | TAACTATCTGACCCTCCTGCCA |
|  |  | 5R |  | gtcagcggccgcatccctgc ATTATTACGGGTCCTGCGGCTAAAT |
|  |  | 3F |  | cacggcgcgcctagcagcgg CCAGTTCTATAGAGGTTTACAATTT |
|  |  | 3R |  | CTCGTGACTTGGACATTCTATC |
|  |  | cF | 1147 | AGGCTACATTTAGCCGCAGG |
|  |  | cR |  | CCGACAAATTGTAAACCTCT |
|  |  | incF | 1066 | TACACCACCCAACATCCTTT |
|  |  | incR |  | TTGCCTTCTGTTGCATTCTT |
| ∆*afl* | AFLA_139200 − AFLA_139440 | 5F |  | AGCCTCTGAACCTTCCAGTCAATACT |
|  |  | 5R |  | gtcagcggccgcatccctgcAAAATGTGAAACTGTTTAGATCGCCT |
|  |  | 3F |  | cacggcgcgcctagcagcggCCTGTGGTGATATTGATGATCCAAGA |
|  |  | 3R |  | GGACTGGTCTGAAACAGTATTACCTC |
|  |  | cF | 61722 | TGAACCCAGGTTATGTAGAAGG |
|  |  | cR |  | TACAAGGGTCAATTAGACAGGC |
| *pyrG* revertant | AFLA_046650 | F |  | CAATAGACCAGTAACGTGTGCAGG |
|  |  | R |  | GTCACGTTCTAAGCTTATCAGCTG |
|  |  | cF | 813 | GTACAGCTAGTGGGTCATAGAGGTAC |
|  |  | cR |  | GTTCAAATATGAAGCCGACGAGCATC |
| - | *pyrG* | pyrG-F |  | ccgctgctaggcgcgccgtgAGCCAGCTAGCTCAGTCTTACCC |
|  |  | pyrG-R |  | gcagggatgcggccgctgacTCGTTCAGAGCTGGTCACAATAA |

Three clones were prepared and assayed for each mutant except ∆AF_*b*_9230, for which a single clone was obtained.

^1^Sequences in uppercase had homology to the *A. flavus* genome, and those in lowercase are sequences facilitating the adaptor pairing with the flanking region of the target genes and the marker gene *pyrG*.

**Supplementary Data**

**Table S2. The predicted function of *F. verticillioides* genes in the fusaric acid and other two clusters having high scores of MIDDAS-M.**

|  | Cluster | Gene | Predicted function by Blastp^a^ | E-value | Accession No. |  |
| --- | --- | --- | --- | --- | --- | --- |
|  | Fusaric acid | FVEG_12519 | Homoserine *O*-acetyltransferase-like protein | 3e-142 | EGS21926.1 |  |
|  | (Cluster 27) | FVEG_12520 | Inducible nitrate reductase | 3e-24 | EFZ03793.1 |  |
|  |  | FVEG_12521 | Aspartate kinase | 1e-163 | EFQ28090.1 |  |
|  |  | FVEG_12522 | No hit | - | - |  |
|  |  | FVEG_12523 | Polyketide synthase | 0.0 | AAR92213.1 |  |
|  |  | FVEG_12524 | Chlorogenic acid esterase precursor | 2e-106 | XP_001391529.1 |  |
|  |  | FVEG_12525 | Acyl-CoA dehydrogenase | 5e-140 | XP_002372691.1 |  |
|  |  | FVEG_12526 | Beta-lactamase | 8e-39 | YP_004311996.1 |  |
|  |  | FVEG_12527 | Class II aldolase/adducin domain protein | 2e-124 | XP_003189008.1 |  |
|  |  | FVEG_12528 | Zinc-binding dehydrogenase | 2e-87 | EFQ32060.1 |  |
|  |  | FVEG_12529 | *O*-acetylhomoserine (thiol)-lyase-like protein | 0.0 | EGS23195.1 |  |
|  |  | FVEG_12530 | Ochratoxin A non-ribosomal peptide synthetase | 2e-148 | XP_002849661.1 |  |
|  |  | FVEG_12531 | FMN-dependent dehydrogenase family protein | 7e-105 | XP_002379238.1 |  |
|  |  | FVEG_12532 | No hit | - | - |  |
|  |  | FVEG_12533 | Major facilitator superfamily transporter | 3e-177 | EFQ27097.1 |  |
|  |  | FVEG_12534 | C6 transcription factor | 1e-31 | EEQ92085.1 |  |
|  |  | FVEG_12535 | *O*-methyltransferase | 1e-72 | XP_001398534.1 |  |
|  | *y1* | FVEG_06988 | No hit | - | - |  |
|  |  | FVEG_06989 | No hit | - | - |  |
|  |  | FVEG_06990 | No hit | - | - |  |
|  | *y2* | FVEG_08709 | Lipase | 6e-131 | XP_002847306.1 |  |
|  |  | FVEG_08710 | ABC transporter | 6e-34 | ABN41482.1 |  |
|  |  | FVEG_08711 | NRPS-like enzyme | 3e-38 | XP_002384551.1 |  |
|  |  | FVEG_08712 | Siderophore iron transporter *mirB* | 0.0 | XP_001940863.1 |  |

*^a^* Top hit with a typical annotation other than “hypothetical protein” or “conserved hypothetical protein”.


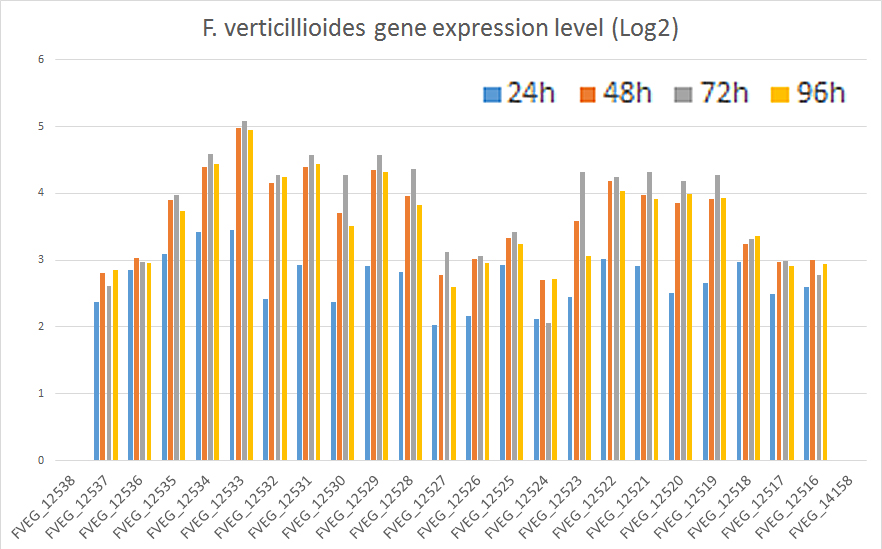


**Reference**

**MIDDAS-M**

**Figure S2. Gene expression values of *F. verticillioides* around the fusaric acid biosynthesis gene cluster.** The values are in log2 scale from time-series of the microarray data, GSE16900.

**Table S3. Number of gene clusters and their member genes detected by MIDDAS-M in *A. flavus*.**

|  |  |  | Number |  |  |
| --- | --- | --- | --- | --- | --- |
|  | Strain | Area | Cluster | Gene |  |
|  | *A. flavus* | All | 240 | 696 |  |
|  | (≥1,016.7) | SB | -*^a^* | 267 |  |
|  |  | NSB | -*^a^* | 702 |  |
|  |  | SMURF+ | 27 | 181 |  |
|  |  | SMURF- | 213 | 788 |  |

*^a^* Values are not evaluated for clusters because SBs and NSBs are defined for genes.

**
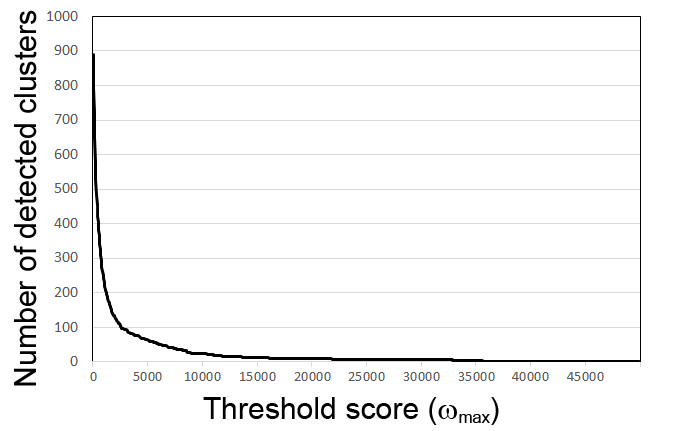
**

**Figure S3. Dependence of the number of unique clusters detected by MIDDAS-M on the threshold score of ω_max_ in *A. flavus*.** A total of 920 unique clusters identified without a threshold decrease exponentially according to the threshold score of ω_max_; 240 at the score of 1,016.7 (0.05 false-positive probability), 64 at 5,000, 24 at 10,000, and 10 at 18,500.


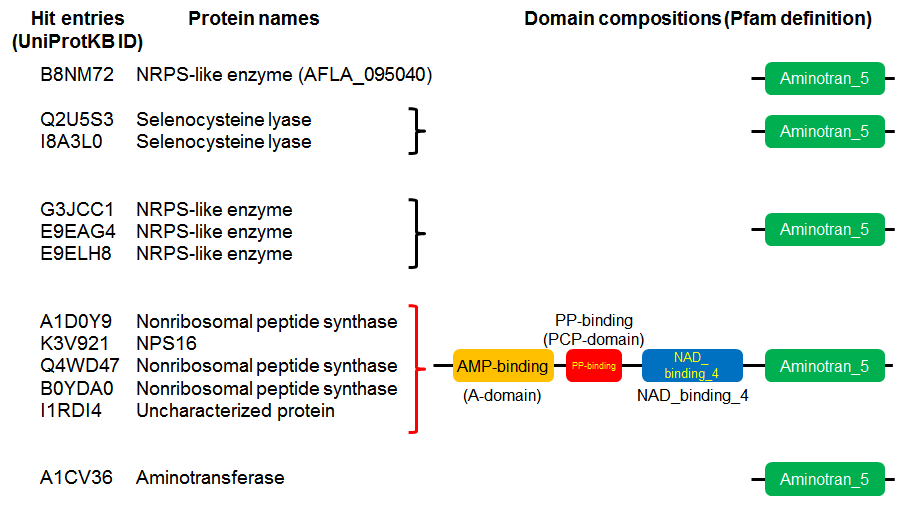


**Figure S4. Top hit proteins by BLAST search of AFLA_095040 against UniProtKB.** The gene AFLA_095040 is annotated as “NRPS-like” in the NCBI database. However, this gene does not include any catalytic domains (A, C, PCP, or TE), according to the Pfam domain definition. E-values for the top hit proteins are smaller than 1.0e-123.

**References**

1. Ma LJ, van der Does HC, Borkovich KA, Coleman JJ, Daboussi MJ, et al. (2010) Comparative genomics reveals mobile pathogenicity chromosomes in *Fusarium*. Nature 464: 367-373.

2. Machida M, Asai K, Sano M, Tanaka T, Kumagai T, et al. (2005) Genome sequencing and analysis of *Aspergillus oryzae*. Nature 438: 1157-1161.

3. Altschul SF, Gish W, Miller W, Myers EW, Lipman DJ (1990) Basic local alignment search tool. J Mol Biol 215: 403-410.

4. Terabayashi Y, Sano M, Yamane N, Marui J, Tamano K, et al. (2010) Identification and characterization of genes responsible for biosynthesis of kojic acid, an industrially important compound from *Aspergillus oryzae*. Fungal Genet Biol 47: 953-961.

5. Georgianna DR, Fedorova ND, Burroughs JL, Dolezal AL, Bok JW, et al. (2010) Beyond aflatoxin: four distinct expression patterns and functional roles associated with *Aspergillus flavus* secondary metabolism gene clusters. Molecular Plant Pathology 11: 213-226.

6. Barrett T, Wilhite SE, Ledoux P, Evangelista C, Kim IF, et al. (2013) NCBI GEO: archive for functional genomics data sets--update. Nucleic Acids Res 41: D991-995.

7. Barrett T, Troup DB, Wilhite SE, Ledoux P, Evangelista C, et al. NCBI GEO: archive for functional genomics data sets--10 years on. Nucleic Acids Res 39: D1005-1010.

8. Team RDC (2008) R: A language and environment for statistical computing. Vienna, Austria: R Foundation for Statistical Computing.

9. Tatusov RL, Koonin EV, Lipman DJ (1997) A genomic perspective on protein families. Science 278: 631-637.

10. Tatusov RL, Fedorova ND, Jackson JD, Jacobs AR, Kiryutin B, et al. (2003) The COG database: an updated version includes eukaryotes. BMC Bioinformatics 4: 41.

11. Min Y, Lv HP, Zheng YH (2007) Fusion PCR-targeted *tylCV* gene deletion of *Streptomyces fradiae* for producing desmycosin, the direct precursor of tilmicosin. Process Biochemistry 42: 729–733.

12. Szewczyk E, Nayak T, Oakley CE, Edgerton H, Xiong Y, et al. (2006) Fusion PCR and gene targeting in *Aspergillus nidulans*. Nature Protocols 1: 3111-3120.

13. Tamano K, Satoh Y, Ishii T, Terabayashi Y, Ohtaki S, et al. (2007) The *β*-1,3-exoglucanase gene *exgA* (*exg1*) of *Aspergillus oryzae* is required to catabolize extracellular glucan, and is induced in growth on a solid surface. Biosci Biotechnol Biochem 71: 926-934.

14. Frisvad JC (1987) High-performance liquid chromatographic determination of profiles of mycotoxins and other secondary metabolites. J Chromatogr 392: 333-347.

15. He ZM, Price MS, Obrian GR, Georgianna DR, Payne GA (2007) Improved protocols for functional analysis in the pathogenic fungus *Aspergillus flavus*. BMC Microbiol 7: 104.
